# The role of monoclonal antibodies against IL-6 or IL-6R in the treatment of thyroid eye disease

**DOI:** 10.1007/s11154-025-10014-5

**Published:** 2026-02-16

**Authors:** Daniel G. Ezra, Atsushi Azumi, César A. Briceño, Fatemeh Rajaii, Mario Salvi, Marco Sales-Sanz, Laura Brockwell, Oluwatobi O. Idowu

**Affiliations:** 1 Moorfields Eye Hospital NHS Foundation Trust, 162 City Road, EC1V 2PD London, UK; 2 School of Health and Medical Sciences, City St George’s, City University, University of London, Northampton Square, London, UK; 3 Ophthalmology Department and Eye Center, Kobe Kaisei Hospital, Kobe, Hyogo Japan; 4 Department of Ophthalmology, Scheie Eye Institute, Perelman School of Medicine, University of Pennsylvania, Philadelphia, PA USA; 5 Department of Ophthalmology, Wilmer Eye Institute, Johns Hopkins University School of Medicine, Baltimore, MD USA; 6 Endocrinology Unit, Graves’ Orbitopathy Center, Fondazione IRCCS Ca’ Granda Ospedale Maggiore Policlinico, Milan, Italy; 7 Department of Ophthalmology, Hospital Universitario Ramon y Cajal, Madrid, Spain; 8 IMO Madrid, Grupo Miranza, Madrid, Spain; 9 Roche Products Ltd., Welwyn Garden City, UK; 10 Genentech, Inc., South San Francisco, CA USA

**Keywords:** Interleukin 6, Thyroid eye disease, Graves’ orbitopathy, Graves’ ophthalmopathy, Pharmacotherapy, Thyroid-associated orbitopathy

## Abstract

Thyroid eye disease (TED) is a chronic inflammatory autoimmune disorder characterized by expansion and fibrosis of orbital tissues, leading to eyelid retraction, proptosis, periorbital edema, restricted ocular motility, corneal ulceration, and (rarely) compressive optic neuropathy. The relationship between TED pathogenesis and interleukin 6 (IL-6)-mediated signaling has created considerable interest in the therapeutic potential of monoclonal antibodies (mAbs) against interleukin 6 (IL-6) or its receptor (IL-6R). A randomized controlled trial (RCT) and several non-RCTs have reported clinical benefits from off-label use of the anti–IL-6R mAb, tocilizumab, in terms of disease activity, proptosis, thyroid-stimulating autoantibody titers (a key trigger for TED), quality of life, and possibly diplopia. Compared with recommended first-line treatment options for TED (high-dose glucocorticosteroids, and in some cases teprotumumab, a mAb against insulin-like growth factor 1 receptor), tocilizumab is associated with fewer adverse events and considerably lower risk of TED relapse following treatment discontinuation. Furthermore, most TED treatment options involve intravenous infusion, whereas tocilizumab can be administered by subcutaneous injection and thereby reduce treatment burden on patients and healthcare systems. Ongoing RCTs evaluating the efficacy and safety of long-acting anti–IL-6/IL-6R mAbs should provide evidence for additional potential reductions in treatment burden. This review aims to provide an overview of IL-6–mediated signaling in TED pathogenesis, evaluate the evidence supporting the use of anti–IL-6/IL-6R mAbs in individuals with TED, and compare the potential benefits and limitations associated with different anti–IL-6/IL-6R mAbs.

**Graphical Abstract:**

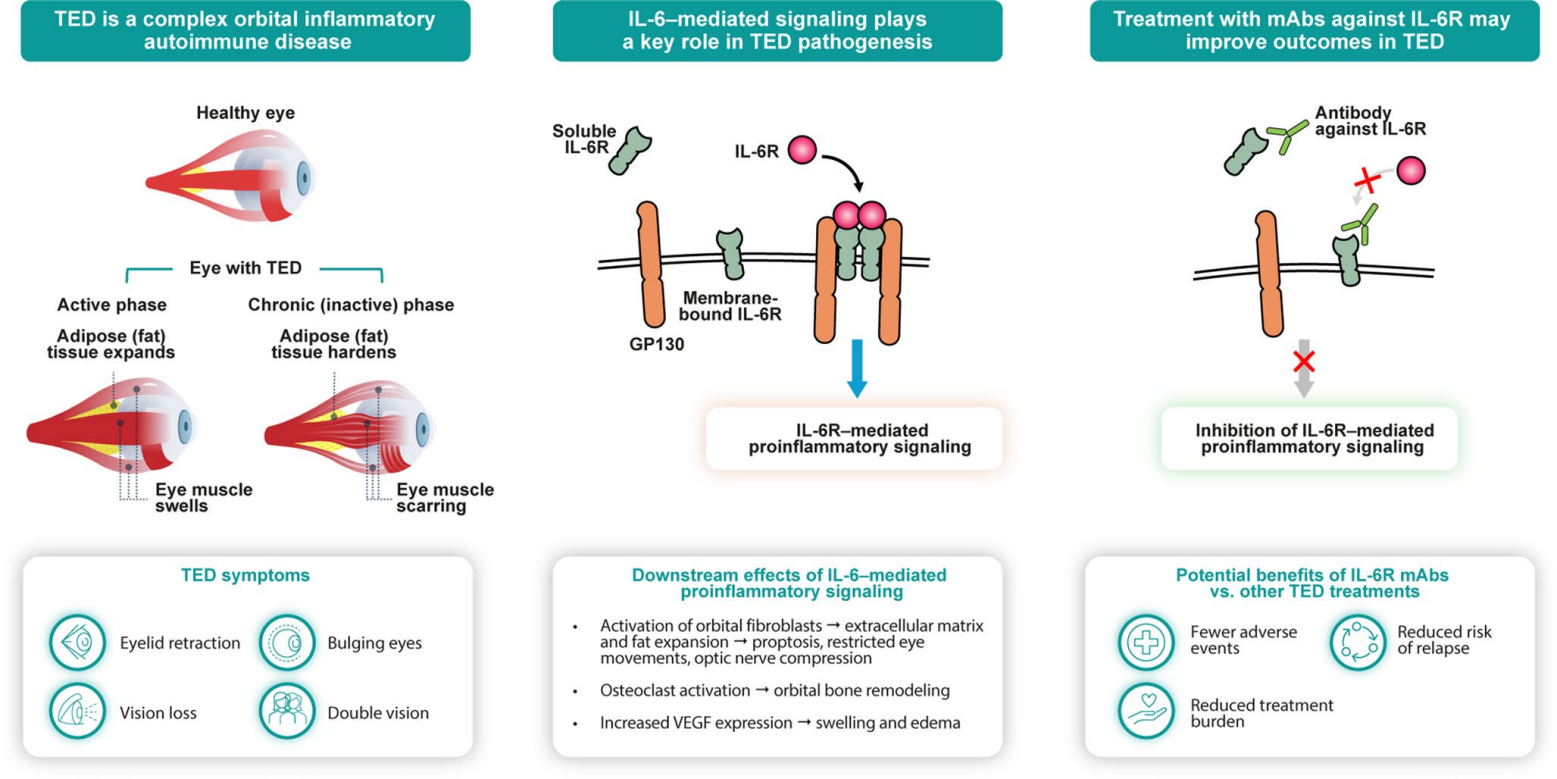

**Supplementary Information:**

The online version contains supplementary material available at 10.1007/s11154-025-10014-5.

## The burden of thyroid eye disease

Thyroid eye disease (TED; also called Graves’ orbitopathy, Graves’ ophthalmopathy, and thyroid-associated ophthalmopathy) is a chronic inflammatory autoimmune disorder that predominantly affects individuals with autoimmune hyperthyroidism, most of whom have Graves’ disease, although it may also occur in those who with hypothyroidism or euthyroidism [1, 2]. The reported prevalence of TED is considerably higher in women (0.12%) than in men (0.04%) [3]. Other risk factors for TED include smoking, uncontrolled thyroid disease, oxidative stress, radioablative iodine therapy, and elevated serum titers of autoantibodies against the thyrotropin receptor [1]. TED is characterized by eyelid retraction, proptosis (eye protrusion), periorbital edema, restricted ocular motility resulting in diplopia, and in rare cases, corneal ulceration and compressive optic neuropathy potentially leading to permanent loss of vision [4–8]. The condition causes the expansion and eventual fibrosis of orbital fat and extraocular muscles, as well as changes in other adnexal structures such as the eyelids and conjunctivae, which further contribute to facial disfigurement [1, 4–6, 9–11]. TED is a highly debilitating, typically bilateral, and often asymmetric condition that affects between 90 and 300 per 100,000 people worldwide [1, 7, 12]. Moderate-to-severe TED, and even cases of mild disease, are associated with a significant reduction in quality of life [13–15].

In the majority of clinical cases, TED and thyroid dysfunction present within 18 months of each other, with TED occurring either before or after the onset of endocrine symptoms [16]. Progression of TED has traditionally been thought to follow two distinct self-limiting phases: an initial active inflammatory phase characterized by orbital tissue changes; and a chronic inactive phase that typically begins 12–18 months after disease onset, during which signs may improve but not fully resolve, with residual disease potentially requiring surgical treatment [12, 17, 18]. Disease activity in TED can be assessed using a modified version of the Clinical Activity Score (CAS), which evaluates inflammatory signs such as pain, redness, and swelling. A CAS of at least 3/7 at initial examination and 4/10 at follow-up examination is suggestive of active disease [1, 4]. The CAS was developed to predict response to treatment, in particular steroid treatment, and although it has a positive predictive value of 80%, its negative predictive value is only 64%, and therefore, CAS assessments should be interpreted with caution [8, 19, 20]. VISA (vision, inflammation, strabismus, and appearance) is another grading system that can be used to assess disease activity and severity in TED [8], but this is less frequently used in clinical trials. TED can also be classified according to its severity using the European Group On Graves’ Orbitopathy (EUGOGO) system [4]. By this system, most cases of TED are mild, with moderate-to-severe disease and vision-threatening disease occurring in approximately 6% and 0.5% of individuals with Graves’ disease, respectively [1, 4, 12].

## Current strategies for clinical management of TED

The clinical management of TED depends on the extent of disease activity and severity, as well as the impact on an individual’s quality of life. Current guidelines from the American Thyroid Association (ATA), European Thyroid Association (ETA), and EUGOGO recommend conservative treatment for all patients, which includes treatment for dry eyes (e.g., artificial tears, gels, ointments, eye masks), dark glasses for dry eye–related photophobia, lifestyle modifications (e.g., smoking cessation, avoidance of passive smoking), and treatment of the underlying thyroid dysfunction [1, 4]. Patients with active, mild TED require close follow-up and, although most cases resolve spontaneously without treatment, a 6-month course of selenium supplementation, while not universally utilized, may be used to improve clinical manifestations and reduce the risk of progression to severe disease [1, 4]. Patients with active, moderate-to-severe TED should be treated with therapies that reduce inflammation and slow the progression of clinical signs during the active phase, with many patients requiring corrective surgery (orbital decompression surgery, strabismus surgery, or eyelid surgery) during the inactive phase [1, 4]. EUGOGO guidelines recommend first-line treatment with high-dose glucocorticosteroids, typically intravenous methylprednisolone [1], whereas the ATA and ETA recommend treating patients according to individual goals [4]. This should include intravenous glucocorticosteroids in patients with signs of disease activity and no significant proptosis or diplopia, and teprotumumab (a monoclonal antibody [mAb] against insulin-like growth factor 1 [IGF-1] receptor [IGF-1R]) in those with significant proptosis and/or diplopia [4]. These sets of guidelines also recommend second-line treatment with immunosuppressive agents such as rituximab (a mAb against CD20), a second course of glucocorticosteroids (intravenous methylprednisolone or oral prednisone/prednisolone with mycophenolate, cyclosporine, or azathioprine), tocilizumab (a mAb against interleukin 6 [IL-6] receptor [IL-6R]), teprotumumab, and/or orbital radiotherapy [1, 4].

Although effective, the long-term use of glucocorticosteroids is limited by a relatively high risk of adverse events (AEs), including new-onset diabetes mellitus, hypertension, acute heart failure, acute liver failure, weight gain, and osteoporosis (Table 1) [21]. Furthermore, 20–30% of patients have an insufficient response to glucocorticosteroid treatment [22], up to 40% suffer TED relapse after glucocorticosteroid discontinuation [4], and 6% may progress to dysthyroid optic neuropathy despite treatment [23]. Similarly, the use of rituximab is limited by the risk of AEs and the need for early treatment due to this agent’s inability to prevent or reverse pathological changes caused by TED [24]. While generally effective for the treatment of diplopia and ocular motility, orbital radiotherapy has limited effects on CAS, proptosis, or eyelid swelling, and is associated with a potential risk of cataract and radiation retinopathy [25]. Although teprotumumab (currently the only on-label treatment for TED [26]) has demonstrated the potential to reduce CAS, proptosis, and diplopia in patients with active [6, 27–31] or chronic TED [32–34], the use of anti–IGF-1R therapy is limited by a high risk of TED relapse following treatment discontinuation. Relapse rates of approximately 29% have been reported in extension studies following randomized controlled trials (RCTs) [29], with rates up to 65% observed in some real-world cohorts after 1 year [35–37]. Teprotumumab is also associated with an increased risk of side effects, including hyperglycemia, hearing impairment (including permanent hearing loss), muscle spasms, nausea, diarrhea, alopecia, fatigue, exacerbation of inflammatory bowel disease, and menstrual irregularities [25, 26, 38–42]. These issues highlight the unmet need for durable and safe treatments that can modify the natural course of active TED and either reverse or reduce the risk of its complications without the need for surgery. In addition, there remains a need for sustainable, long-term treatment options that are safe and effective in the chronic phase of TED and can reduce the risk of relapse, alleviate patient anxiety surrounding disease progression, and preserve quality of life.

**Table 1:** Pharmacotherapies recommended for the treatment of active, moderate-to-severe TED

Characteristic	Pharmacotherapies recommended by ATA, ETA, and EUGOGO [1, 4, 133]						
	Immunotherapy				Rituximab(anti-CD20 mAb)	Teprotumumab (anti–IGF-1R mAb)	Tocilizumab (anti–IL-6R mAb)
	IV GC	Oral GC	Mycophenolate mofetil	Mycophenolate mofetil + GC			
Dose and route of administration [4]	IV 0.5 g Q1W for 6 weeks, followed by 0.25 g Q1W for 6 weeks	PO daily for 3 months (tapering from prednisolone 100 mg daily; cumulative dose 4 g)	PO 0.72 g daily for 24 weeks	Mycophenolate mofetil 0.72 g/day for 6 weeks + IV 0.5 g GC/Q1W for 6 weeks	IV 1 g Q1W for 2 weeks IV 0.5 g–0.1 g single dose	IV Q3W for 6 months (first dose 10 mg/kg, subsequent doses 20 mg/kg)	IV 8 mg/kg Q4W for 12 weeks
Percentage of patients with beneficial effect ^**a**^							
Composite outcomes	23–53	Not reported	Not reported	63	8–60	74	73
CAS	45–83			80	31–100	62	93
Proptosis	0–46			0	0	77	27
Diplopia	0–19			0	0	70	7
AEs affecting ≥ 10% of patients [4]	Hyperglycemia	Hyperglycemia, GI symptoms, weight gain, Cushingoid facies	Not applicable	GI symptoms	Infusion reactions	Hyperglycemia, GI symptoms, myalgias, alopecia, fatigue	Fatigue, hyperlipidemia, neutropenia
AEs affecting ≥ 5% of patients [4]	GI symptoms	Hypertension	Not applicable	Not applicable	GI symptoms	Dry skin, taste disturbance	Pruritus
Severe AEs [4]	Infection (≥ 5%)	Infection (≥ 5%)	Infection, hepatitis	Infection (≥ 10%)	Transient vision loss (≥ 5%)	Hearing loss, inflammatory bowel disease exacerbation (≥ 10%)	Infection (≥ 10%), hepatitis (≥ 5%)
**Relapse rate after treatment discontinuation**	21–40% at week 12 [4]	11% at week 24 [4]	Not reported	Up to 11% at week 24 [4]	0–15% at week 40 [4]	Up to 65% at 1 year [35–37]	8.2% [45]

^a^Comparisons between treatments should be interpreted with caution due to differences in patient populations, assessments, endpoint definitions, and treatment durations across studies. The definition of “composite outcome” varies and is based on a combination of activity and severity scores. Proptosis improvements were defined in the majority of studies as a reduction ≥ 2 mm. Diplopia was assessed using the Gorman scoring system

AE: adverse event; ATA: American Thyroid Association; CAS: Clinical Activity Score; ETA: European Thyroid Association; EUGOGO: European Group On Graves’ Orbitopathy; GC: glucocorticosteroid; GI: gastrointestinal; IGF-1R: insulin-like growth factor 1 receptor; IL-6R: interleukin 6 receptor; IV: intravenous; mAb: monoclonal antibody; PO: per os; Q1W: once weekly; Q4W: every 4 weeks; TED: thyroid eye disease

Recent evidence for a relationship between IL-6–mediated signaling and TED pathogenesis [5, 9–11, 43] has led to considerable interest in the potential use of IL-6/IL-6R inhibitors for the treatment of TED. Although the safety and efficacy of mAbs against IL-6 or IL-6R have been demonstrated in a wide range of inflammatory conditions [44, 45], they are not licensed for use in individuals with TED due to the limited number of RCTs in this setting [1, 21, 46]. Based on results from an RCT in individuals with active, glucocorticosteroid-resistant, moderate-to-severe TED [47], the ATA, ETA, and EUGOGO all recommend the off-label use of tocilizumab as a second-line treatment option [1, 4]. In this review, we aim to provide an overview of IL-6–mediated signaling in TED pathogenesis, evaluate the clinical evidence supporting the use of anti–IL-6/IL-6R mAbs in individuals with TED, and compare the potential benefits and limitations associated with different anti–IL-6/IL-6R mAbs.

## Intracellular signaling pathways activated by the binding of IL-6 to IL-6R

**Fig. 1:**
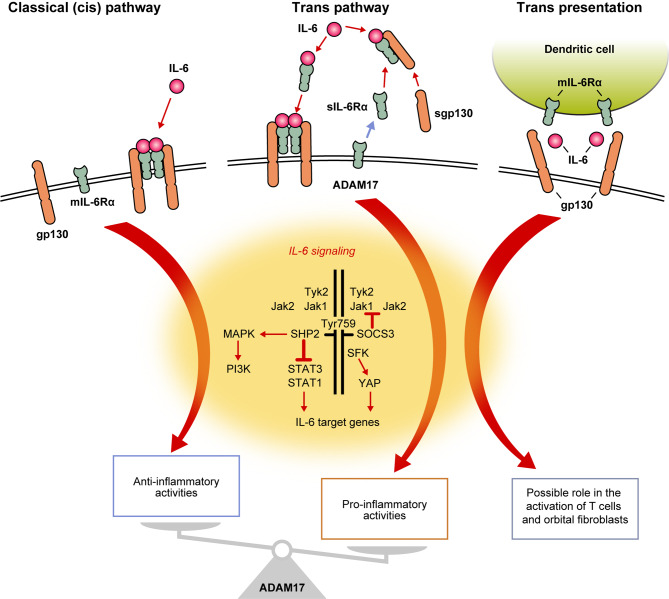
The three different IL-6–mediated signaling pathways activated via IL-6R. Based on Avci AB, Feist E, Burmester GR. BioDrugs. 2024;38(1):61–71. ADAM17: a disintegrin and metalloprotease domain 17; gp: glycoprotein; IL-6: interleukin 6; IL-6R: interleukin 6 receptor; Jak: Janus kinase; MAPK: mitogen-activated protein kinases; mIL-6Rα: membrane-bound interleukin 6 receptor; PI3K: phosphoinositide-3 kinases; sgp130: soluble gp130; sIL-6Rα: soluble interleukin 6 receptor; SFK: Src family kinases; SHP: SH2-containing protein tyrosine phosphatase; SOCS: suppressor of cytokine signaling; STAT: signal transducer and activator of transcription factor; Tyk: tyrosine kinase; Tyr: tyrosine; YAP: Yes-associated protein

Human IL-6 is a 212-amino acid, pleiotropic cytokine that plays a fundamental role in a wide range of physiologic processes, including innate and adaptive immune responses, inflammation, fibrosis, acute phase responses, and metabolic reactions [48–52]. IL-6 is produced by numerous cell types (e.g., monocytes, macrophages, T cells, adipocytes, fibroblasts, endothelial cells) in response to tissue damage or infection and circulates around the body within the bloodstream [53, 54]. Circulating IL-6 activates intracellular signaling pathways by binding to IL-6R, which is a protein complex comprised of an 80-kDa, IL-6–binding receptor molecule (IL-6Rα) and transmembrane gp130 [55, 56]. IL-6Rα exists in two forms: membrane-bound (mIL-6Rα) and soluble (sIL-6Rα), the latter of which is proteolytically cleaved from cell membranes by a disintegrin and metalloprotease domain 17 (ADAM17; Fig. 1) [56, 57]. The classical cis-signaling pathway is activated when mIL-6Rα binds circulating IL-6 and initiates signaling via the cytoplasmic domain of gp130, whereas the trans-signaling pathway is activated when sIL-6Rα binds circulating IL-6 and this complex subsequently interacts with transmembrane gp130 [57, 58]. A third pathway, referred to as the “trans-presentation” or “cluster signaling” pathway, can be activated when a cell expressing mIL-6Rα “presents” IL-6 bound on its surface to a different cell that expresses transmembrane gp130. Irrespective of the pathway activated, the IL-6–induced signal results in activation of Janus kinase (JAK) 1, JAK2, and tyrosine kinase 2 associated with the cytoplasmic domain of gp130. The subsequent recruitment and phosphorylation of signal transducer and activator of transcription (STAT) 3 and STAT1 leads to these transcription factors translocating to the nucleus, where they modulate the expression of specific genes, with additional signaling occurring via mitogen-activated protein kinases and phosphoinositide-3 kinases. IL-6–induced expression of suppressor of cytokine signaling 3 (SOCS3; a potent negative regulator of the JAK/STAT pathway) terminates the IL-6–mediated signaling cascades via a feedback loop. The downstream effects of IL-6–mediated signaling vary between the three pathways [57, 58]. For example, activation of the cis-signaling pathway predominantly results in effects that contribute to acute phase responses, protection against bacterial infections, and anti-inflammatory effects, whereas activation of the trans-signaling pathway is predominantly associated with proinflammatory effects. Given this distinction, therapeutic agents that inhibit IL-6R may provide broader control of inflammation and tissue remodeling in TED by blocking both cis- and trans-signaling, rather than agents targeting IL-6 alone. A recent review by Murdock et al. (2025) has further reinforced the importance of IL-6 signaling in TED pathogenesis, providing evidence in support of therapies targeting IL-6/IL-6R to improve outcomes [59].

## The role of IL-6–mediated signaling pathways in autoimmune and inflammatory disorders

**Fig. 2:**
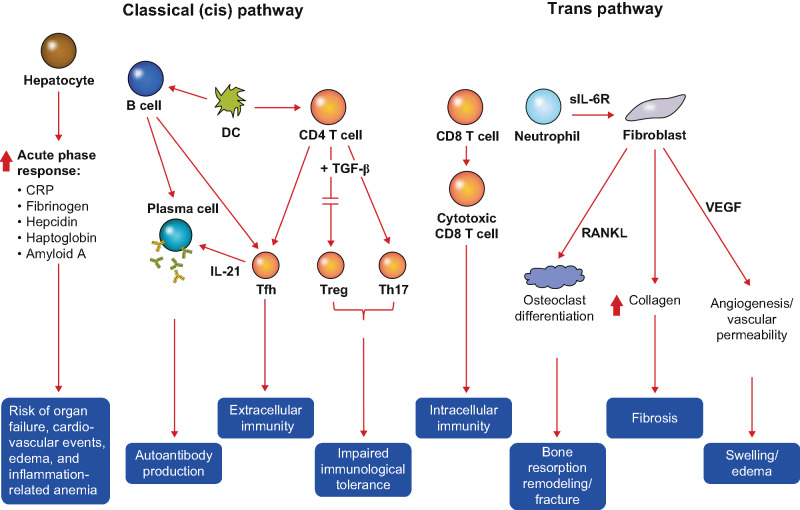
The role of IL-6 cis- and trans-signaling pathways in autoimmune and inflammatory disorders. Created with reference to: Yao X, Huang J, Zhong H, et al. Pharmacol Ther. 2014;141(2):125–39. CRP: C-reactive protein; DC: dendritic cell; IL: interleukin; sIL-6R: soluble interleukin-6 receptor; TGF-β: transforming growth factor beta; Th17: T helper 17 cell; Treg: regulatory T cell; Tfh: T follicular helper cell; VEGF: vascular endothelial growth factor

Naïve CD4 + T cells express high levels of both IL-6Rα and gp130 and are highly sensitive to circulating IL-6 concentrations [54, 60, 61]. Low concentrations of IL-6 in combination with transforming growth factor beta (TGF-β) promote the differentiation of naïve CD4 + T cells into anti-inflammatory regulatory T (Treg) cells, whereas high concentrations of IL-6 in combination with TGF-β promote the differentiation of T cells into proinflammatory T helper 17 (Th17) cells. In healthy individuals, high concentrations of IL-6 also result in the downregulation of IL-6Rα expression in T cells and a decreased proinflammatory response. In autoimmune and inflammatory conditions such as TED, chronic overexpression of IL-6 results in the dysregulation of IL-6–mediated signaling and impaired immunologic tolerance due to an imbalance between the activity of Treg and Th17 cells (Fig. 2) [54]. This imbalance can be exacerbated by IL-6–mediated increases in the expression of immunoglobulin-regulating IL-21, differentiation of CD8 + T cells into cytotoxic T cells, and differentiation of activated B cells into antibody-producing plasma cells.

Increased concentrations of IL-6 can also trigger potentially pathologic processes through interactions with IL-6R expressed on other cell types [54, 62]. For example, binding of circulating IL-6 to IL-6R expressed on hepatocytes stimulates the expression of acute phase proteins (e.g., C-reactive protein, serum amyloid A, fibrinogen, α1-antichymotrypsin) while decreasing expression of fibronectin, albumin, and transferrin. Such changes can increase the risk of organ failure, cardiovascular events, edema, and inflammation-related anemia. Binding of IL-6 to IL-6R expressed on fibroblasts can stimulate the receptor activator of nuclear factor-kappa B ligand (RANKL)-mediated differentiation and activation of osteoclasts, potentially leading to bone resorption, remodeling, and fractures. IL-6–mediated signaling can also increase fibroblast production of collagen, which increases the risk of fibrosis, as well as increase expression of vascular endothelial growth factor (VEGF), resulting in swelling and edema due to enhanced angiogenesis and vascular permeability. Many of these features can be observed in individuals with TED [54, 63].

## The role of IL-6–mediated signaling in the pathophysiology of TED

**Fig. 3:**
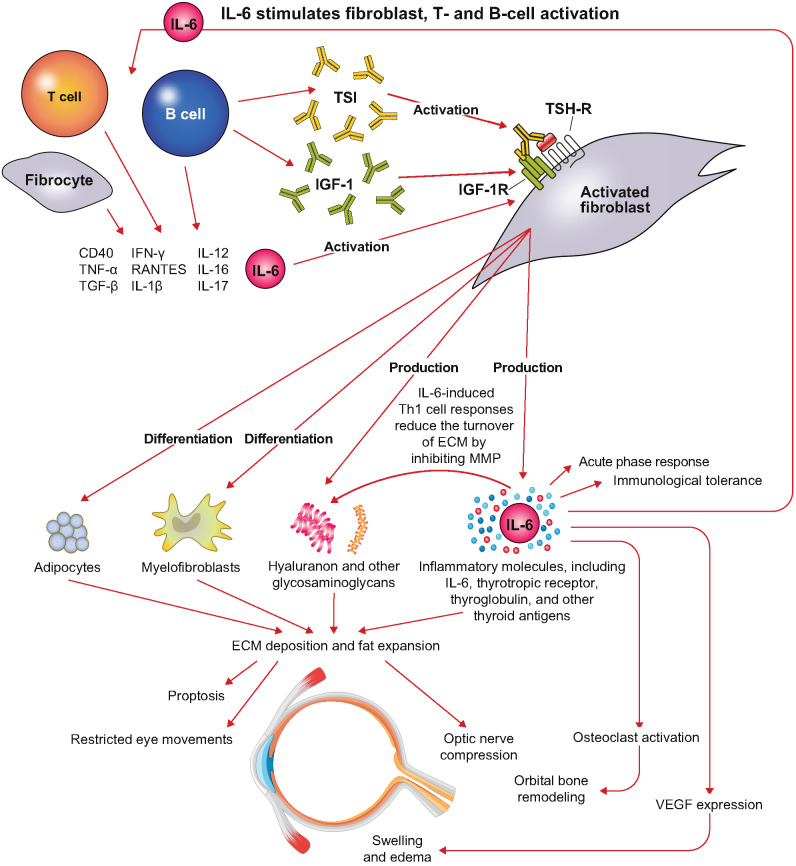
Fig. 3 The role of IL-6 in TED pathogenesis. Created with reference to: Smith TJ. Front Endocrinol (Lausanne). 2020;11:610337, and Yu CY, Ford RL, Wester ST, Shriver EM. Indian J Ophthalmol. 2022;70(7):2335–45. ECM: extracellular matrix; IGF-1: insulin-like growth factor 1; IGF-1R: insulin-like growth factor 1 receptor; IL: interleukin: IFN-γ: interferon gamma; MMP: matrix metalloproteinase; RANTES: regulated upon activation, normal T cell expressed and secreted; TED: thyroid eye disease; TGF-β: transforming growth factor beta; Th1: T helper 1; TNF-α: tumor necrosis factor alpha; TSH-R: thyroid-stimulating hormone receptor; TSI: thyroid-stimulating immunoglobulin; VEGF: vascular endothelial growth factor

IL-6–mediated signaling plays a key role in the pathophysiology of TED, and IL-6 itself has been proposed as a biomarker for evaluating the inflammatory activity of the disease [64–66]. The autoimmune process leading to TED begins with the infiltration of fibrocytes and activated B and T cells into the orbital connective tissue and extraocular muscles (Fig. 3) [5, 9–11, 43, 61]. These infiltrating cells express an array of proinflammatory cytokines (e.g., IL-1β, IL-6, IL-8, IL-16, tumor necrosis factor alpha [TNF-α], regulated upon activation, normal T cell expressed and secreted [RANTES], CD40 ligand) that promote activation of orbital fibroblasts and induce them to overexpress thyroid-stimulating hormone receptor (TSH-R) and IGF-1R. Stimulation of the induced TSH-R by circulating thyroid-stimulating autoantibodies, and potentially the stimulation of induced IGF-1R by anti–IGF-1R autoantibodies, drives orbital fibroblast proliferation and differentiation into myofibroblasts and adipocytes, as well as increasing the synthesis of glycosaminoglycans and further increasing expression of proinflammatory cytokines, TSH-R, thyroglobulin, and other thyroid antigens. Overall, these processes lead to high levels of deposition of extracellular matrix and expansion of adipose tissue, resulting in restricted eye movement, proptosis, and optic nerve compression due to orbital tissue enlargement, congestion, and inflammation of extraocular muscles.

When activated by IL-1β, orbital fibroblasts express high levels of IL-6 due to enhanced IL-6 gene promoter activity and enhanced stability of IL-6 messenger RNA (mRNA) transcripts [50]. This exacerbates the ongoing autoimmune process by increasing the expression of TSH-R in these cells (Fig. 3) [5, 9–11, 43]. The quantity of extracellular matrix increases due to an IL-6–mediated shift from an acute inflammatory state to a more chronic, profibrotic state via the induction of Th1 responses that inhibit metalloprotease activity and reduce the turnover of extracellular matrix components [67]. In addition to driving inflammation, IL-6 signaling directly contributes to fibrosis by promoting orbital fibroblast activation, myofibroblast differentiation, and extracellular matrix deposition [11, 59]. These profibrotic effects may contribute to tissue remodeling in TED, including extraocular muscle fibrosis and restricted ocular motility. Symptoms of TED can also be exacerbated by binding of IL-6 to IL-6R expressed on orbital fibroblasts, which can lead to swelling and edema due to increased expression of VEGF [52], as well as bone remodeling due to activation of osteoclasts [63]. The expression of IL-6 by differentiated fibroblasts and adipocytes further stimulates the production of thyroid-stimulating autoantibodies by activated B cells [21]. IL-6 is synthesized by multiple cell types that infiltrate the orbit, including T cells, B cells, macrophages/monocytes, and CD34+ fibrocytes, and is also increased by several upstream triggers [11, 59, 68]. For example, costimulation and crosstalk between CD40 on orbital fibroblasts and its ligand on T cells and B cells increases IL-6 synthesis. In addition, CD34+ orbital fibroblasts express autoantigens, including the TSH-R. Autoantibodies target the TSH-R to activate signaling pathways in orbital fibroblasts and enhance the production of inflammatory cytokines, including IL-6 [11, 59, 68].

Whereas pharmacotherapeutic inhibition IGF-1/IGF-1R–mediated signaling does not appear to induce anti-inflammatory effects beyond reductions in titers of circulating autoantibodies, inhibition of IL-6/IL-6R–mediated signaling has been shown to modulate immunopathogenetic pathways upstream of autoantibody-related effects [46, 54, 69]. For example, IL-6–mediated signaling plays a key role in promoting the activation and differentiation of T cells into proinflammatory subsets and B cells into antibody-producing plasma cells [54, 70], as well as promoting the accumulation of adipocytes and activation of orbital fibroblasts implicated in tissue remodeling and upregulation of TSH-R expression [21, 71–79]. Overall, these observations suggest that the role of IL-6 in TED pathogenesis lies upstream to that of IGF-1 [46, 54, 69, 70].

## The rationale for inhibition of IL-6/IL-6R–mediated signaling in the treatment of TED

Findings from human studies have demonstrated that IL-6 is involved in TED pathogenesis. Expression levels of IL-6 and IL-6R are elevated in individuals with Graves’ disease [80, 81], and individuals with active TED have significantly greater serum concentrations of IL-6 than those without, with these observations being independent of thyroid dysfunction [82]. Furthermore, expression levels or concentrations of serum IL-6R [80, 83], serum IL-6 [64, 83–85], tear fluid IL-6 [86–88], orbital tissue IL-6 [89, 90], and orbital tissue IL-6R [90] are all significantly greater in individuals with active TED compared with those with inactive TED, as well as greater in those with inactive TED compared with those without TED. Exposure to IL-6 is associated with increased expression of the thyroid autoantigen thyrotropin receptor on orbital fibroblasts of individuals with TED [91, 92]. Finally, transcripts of IL-6 mRNA are present in the extraocular muscles and orbital fat of individuals with TED, and these are positively correlated with orbital volume [93].

When taken together, the association between greater expression of IL-6/IL-6R and greater TED activity and severity strongly suggests that therapies capable of antagonizing IL-6/IL-6R–mediated signaling may modify progression of TED. Notably, blocking IL-6R rather than IL-6 alone may provide effective inhibition of local inflammation and tissue remodeling by blocking both cis- and trans-signaling pathways, the latter of which is particularly associated with proinflammatory and fibrotic processes. Specifically, inhibition of IL-6R may help restore the Th17/Treg balance, which in turn can prevent autoantibody formation, B-cell proliferation, and T-cell–driven inflammation, as well as orbital fibroblast signaling and differentiation. This approach can also help to mitigate autoimmune thyrotoxicosis associated with TED [94–96]. Findings from both small randomized clinical trials and real-world use of tocilizumab for TED have shown improvements in CAS and quality of life, reduction in proptosis, diplopia, and thyroid-stimulating immunoglobulin levels, and low relapse rates [6, 47, 97]. Based on these positive results, the EUGOGO 2021 guidelines and the joint ATA and ETA consensus have recommended IL-6 receptor inhibition (tocilizumab) as a second-line treatment option for active, moderate-to-severe TED [1, 4].

## Currently available and investigational anti–IL-6/IL-6R mAbs

The long-term safety and efficacy of currently available and investigational mAbs against IL-6 or IL-6R have been demonstrated in a wide range of inflammatory disorders [44, 45, 54]. For example, tocilizumab is approved for the treatment of moderate-to-severe rheumatoid arthritis, systemic juvenile idiopathic arthritis, polyarticular-course juvenile idiopathic arthritis, adult-onset Still’s disease (Japan), chimeric antigen receptor T cell cytokine release syndrome, multicentric Castelman’s disease (Japan), giant cell arteritis, Takayasu arteritis (Japan), and systemic sclerosis–associated interstitial lung disease (USA); satralizumab is approved for the treatment of neuromyelitis optica spectrum disorders; sarilumab is approved for the treatment of rheumatoid arthritis; and siltuximab is approved for the treatment of multicentric Castelman’s disease (Table 2) [44, 98]. Anti–IL-6R mAbs, such as tocilizumab, satralizumab, sarilumab, vobarilizumab, and levilimab, antagonize all three IL-6–mediated signaling pathways, whereas anti–IL-6 mAbs, such as siltuximab, olokizumab, sirukumab, clazakizumab and pacibekitug only antagonize the cis- and trans-signaling pathways (Fig. 1) [57, 58]. Although the role of the trans-presentation pathway in TED pathophysiology has yet to be elucidated, it is thought that this pathway may involve the activation of T cells and orbital fibroblasts. It is therefore possible that mAbs against IL-6R are more potent than those against IL-6 when used in the setting of TED.

**Table 2:** Characteristics of currently available and investigational anti–IL-6/IL-6R monoclonal antibodies

Monoclonal antibody	Target antigen [44]	Species	Dosing modality	Approved indications	Clinical trials in TED
**Tocilizumab**	IL-6R	Humanized	IV 8 mg/kg Q4W; SC 162 mg Q1–2W [44, 99]	RA, sJIA, pcJIA, AoSD (Japan), MCD (Japan), CRS, GCA, TAK (Japan), SSc-ILD (USA) [44, 99]	Phase 2b TOGO trial (NCT04876534)^a^ Phase 3 [47]
**Satralizumab**	IL-6R	Humanized	SC 120 mg Q2W for three doses, Q4W thereafter	NMOSD [44]	Phase 3 SatraGO-1 and SatraGO-2 trials (NCT05987423; Eudra CT 2023–503309-13)^a^
**Sarilumab**	IL-6R	Human	SC 150 mg, 200 mg Q2W	RA [44]	―
**Vobarilizumab**	IL-6R	Humanized nanobody	SC 75 mg, 150 mg, 225 mg Q2–4 W	―	―
**Levilimab**	IL-6R	Human	SC 162 mg Q1–2W	―	―
**Olokizumab**	IL-6	Humanized [44]	SC 64 mg Q2–4W	RA and COVID-19 (Russia and some Eastern European countries) [44, 134]	―
**Sirukumab**	IL-6	Human	SC 50 mg, 100 mg Q2–4W	―	―
**Clazakizumab**	IL-6	Humanized	SC 25 mg, 100 mg, 200 mg Q4W	―	―
**Siltuximab**	IL-6	Chimeric [44]	IV 11 mg/kg Q3W [44]	MCD (USA/EU) [44]	―
**Pacibekitug**	IL-6	Humanized [44]	SC 20 mg, 50 mg Q8W	―	Phase 2b spiriTED trial (NCT06088979)^a^

^a^Ongoing trial (study not yet completed)

AoSD: adult-onset Still’s disease; COVID-19: coronavirus disease 2019; CRS: cytokine release syndrome; GCA: giant cell arteritis; IL-6: interleukin 6; IL-6R: interleukin 6 receptor; IV: intravenous; MCD: multicentric Castelman’s disease; NMOSD: neuromyelitis optica spectrum disorders; pcJIA: polyarticular-course juvenile idiopathic arthritis; Q1W: once weekly; Q2W: every 2 weeks; Q3W: every 3 weeks; Q4W: every 4 weeks; Q8W: every 8 weeks; RA: rheumatoid arthritis; SC: subcutaneous; sJIA: systemic juvenile idiopathic arthritis; SSc-ILD: systemic sclerosis–associated interstitial lung disease; TAK: Takayasu arteritis; TED: thyroid eye disease

Other key differences between mAbs against IL-6 or IL-6R include their routes of administration and dosing frequencies. With the exception of tocilizumab (administered as an intravenous infusion every 4 weeks or a subcutaneous injection every 1–2 weeks [44, 99]) and siltuximab (administered as an intravenous infusion every 3 weeks [100]), all the other currently available or investigational anti–IL-6/IL-6R mAbs are administered as a subcutaneous injection every 1–2 weeks (sarilumab, levilimab) [101], every 2–4 weeks (vobarilizumab, olokizumab, sirukumab), every 4 weeks (satralizumab, clazakizumab), or every 8 weeks (pacibekitug). Differences in dosing frequency can be important because therapies with longer intervals between doses, and therefore with reduced treatment burden on patients, are often associated with better durability of therapeutic benefits and improved outcomes due to higher rates of treatment adherence/persistence [102]. Among these, vobarilizumab, sirukumab, and clazakizumab are not currently approved for use in any indication and remain under investigation in clinical trials, including but not limited to TED.

## Evidence for the benefits of treatment with anti–IL6/IL-6R mAbs in individuals with TED

### Tocilizumab

Tocilizumab is a humanized anti–IL-6R mAb that binds specifically to both mIL-6Rα and sIL-6Rα, thereby antagonizing all three IL-6–mediated signaling pathways [44, 45]. Despite being recommended as a second-line treatment option for active, moderate-to-severe TED [1, 4], tocilizumab is not licensed for use in this setting [21].

Guidelines recommend tocilizumab as a treatment option for patients with active, moderate-to-severe TED who are resistant to corticosteroids [4, 18]. This recommendation is supported by evidence from one RCT [47] and several non-randomized studies [97, 103–110]. Patients on off-label tocilizumab for TED should be closely monitored throughout the treatment course to manage potential adverse events, which include infection, hyperlipidemia, neutropenia, thrombocytopenia, elevated transaminase levels, and, in rare cases, anaphylaxis or bowel perforation [4]. While high levels of thyroid-stimulating immunoglobulin (TSI) might typically indicate severe disease activity, evidence from multiple studies suggests that elevated TSI levels do not contraindicate tocilizumab therapy [47, 103, 108]. Careful evaluation of each patient’s individual profile is necessary, particularly for those at higher risk of complications (e.g., patients with active infections or pre-existing hepatic conditions), and consistent monitoring is essential during treatment.

Tocilizumab may reduce IL-6R–mediated inflammation that persists in some patients despite corticosteroid treatment by binding both mIL-6R and sIL-6R and inhibiting IL-6 pathway signaling. Results from a prospective in vitro study may provide additional mechanistic context for this [111]. In peripheral blood mononuclear cells (PBMCs) from patients with TED cultured with dexamethasone, both IL-6 and sIL-6R were significantly reduced compared with untreated (basal) cells or PBMCs cultured with tocilizumab. In contrast, tocilizumab significantly increased the sIL-6R level versus basal levels but had no effect on IL-6 levels. This may be due to the formation of sIL-6R/tocilizumab complexes, which could prolong the elimination half-life of sIL-6R while IL-6 is unable to bind to sIL-6R [111]. These findings underscore the distinct pharmacological actions of the two treatments, with corticosteroids reducing gene expression of IL-6 and IL-6R, and tocilizumab specifically blocking the IL-6R and inhibiting IL-6 pathway signaling with higher specificity.

A systematic literature review of studies evaluating tocilizumab in TED identified 29 articles covering 208 participants, including one RCT and several observational studies [45, 106, 108–110, 112]. Since the publication of this systemic literature review in 2024, two additional, albeit small (*N* = 12 and *N* = 19), observational studies have been published [103, 104].

The tocilizumab RCT was a phase 3, double-blind, multicenter study that included 32 individuals with active, moderate-to-severe TED previously treated with glucocorticosteroids. This study found that the proportion of participants with a change from baseline in CAS ≥ 2 points at week 16 (the primary endpoint) was significantly greater in those receiving intravenous tocilizumab every 4 weeks compared with those receiving placebo (93.3 vs. 58.8%; *p* = 0.04) [47]. Compared with placebo at week 16, those receiving tocilizumab were also significantly more likely to achieve inactive disease (CAS < 3, 86.7 vs. 35.2%; *p* = 0.005) and have an improved EUGOGO-proposed composite ophthalmic score (73.3 vs. 29.4%; *p* = 0.03). The reduction in exophthalmos size at week 16 was also significantly greater in those treated with tocilizumab compared with placebo (median [interquartile range]: −1.5 [−2.0 to 0.5] vs. 0.0 [−1.0 to 0.5] mm; *p* = 0.01). Although these results are encouraging, findings from this RCT should be interpreted with caution due to the small number of participants; the inclusion of participants previously treated with glucocorticosteroids; the primary endpoint being based on CAS, which has been shown to have high negative predictive value in subsets of patients who do not follow the standard disease course [19, 20]; and the use of an unvalidated composite ophthalmic score evaluated and analyzed post hoc. This study reported that the significant treatment benefits achieved at week 16 were not maintained through week 40, which may be explained, at least in part, by the variable times since TED diagnosis among the participants. In contrast to more recent studies, this baseline variable did not form part of the inclusion criteria and therefore participants could have been in different phases of the disease. Furthermore, the dosing schedule used in this study was based on prior studies in rheumatoid arthritis and not TED. In the non-RCT studies, treatment with tocilizumab was associated with improvements in both CAS (≥ 2 points) and proptosis [103, 104, 106, 108–110, 112], with some studies [103, 104, 106] (but not others [113, 114]) also reporting improvements in diplopia. A meta-analysis of data from 12 studies including a total of 219 individuals with active TED previously treated with glucocorticosteroids reported a mean reduction in CAS of 4.60 points (95% CI: 3.88–5.32) across 10 studies, and a mean reduction in proptosis of 2.04 mm (95% CI: 1.42–2.65) across seven studies [115]. Importantly, treatment with tocilizumab has also been associated with reductions in titers of thyroid-stimulating immunoglobulins [97, 103, 109, 116, 117], with the meta-analysis reporting a mean reduction of 10.62 IU/L (95% CI: 4.67–10.62) across five studies [115]. This may be explained by IL-6 inhibition by tocilizumab, which dampens B-cell/plasma-cell activation [21], and thus reduces autoantibody production. The disease-modifying effects of tocilizumab have been reported to be similar irrespective of the route of administration [45].Treatment with tocilizumab is generally well tolerated in individuals with TED. No severe AEs were reported in any of the studies, with the most frequent AEs including increases in hepatic enzyme activity, hypercholesterolemia, and transient neutropenia [45]. The tocilizumab RCT reported two serious AEs among the group treated with tocilizumab: one participant diagnosed with latent tuberculosis had a moderate increase in hepatic transaminase activity at week 8, and another participant had acute pyelonephritis at week 30 [47]. Across all the studies that reported rates of TED relapse following discontinuation of tocilizumab, only 12 of 146 participants (8.2%) relapsed, and these occurred between month 1 and month 30 after treatment discontinuation [45, 105, 107, 109, 117–120]. Although definitive conclusions cannot be drawn from cross-trial comparisons, this rate of relapse is lower than the rates of 21–40% observed at week 12 in those discontinuing first-line intravenous glucocorticosteroid therapy [4], is similar to the rate of 0–15% observed at week 40 in those treated with rituximab [4], and is notably lower than the rates of approximately 29% reported in extension studies following RCTs [29] and up to 65% observed after 1 year in those treated with teprotumumab without prior treatment with glucocorticosteroids [4, 35–37].

Evidence suggests that the safety and efficacy of tocilizumab are similar when administered as either an intravenous infusion or subcutaneous injection [45, 97], with intravenous infusions administered every 4 weeks and subcutaneous injections administered every 1–2 weeks [57]. Subcutaneous administration may offer an advantage over current pharmacotherapies recommended for active, moderate-to-severe TED, the majority of which are administered via regular intravenous infusions and impose a high treatment burden [45, 57, 97].

Overall, results obtained from clinical studies support the use of tocilizumab in individuals with active, moderate-to-severe TED, but further, larger randomized trials are required to directly compare its safety and efficacy with other treatment options and to establish whether tocilizumab can control the disease and avoid relapses in those with inactive disease. The phase 2b, single-blind, multicenter Tocilizumab in Active Moderate-Severe Graves’ Orbitopathy (TOGO) trial (NCT04876534) is underway to compare the safety and efficacy of intravenous tocilizumab with the standard of care (intravenous methylprednisolone) in 64 individuals with TED.

### Satralizumab

Satralizumab is a humanized immunoglobulin G2 (IgG2) anti–IL-6R mAb that, similar to tocilizumab, antagonizes binding of IL-6 to both mIL-6Rα and sIL-6Rα [121–123]. Approved for the treatment of adults with anti–aquaporin-4-seropositive neuromyelitis optica spectrum disorders in > 85 countries, the persistent efficacy and favorable long-term safety profile of satralizumab in this setting have been demonstrated using up to 9 years of exposure data obtained from pivotal phase 3, open-label extension, and post-marketing surveillance studies [124–126]. As of May 31, 2024, satralizumab has been prescribed to an estimated 5,703 patients with neuromyelitis optica spectrum disorders worldwide [127]. Satralizumab was designed using pH-dependent recycling antibody technology [128, 129], which is a technique based on the observation that half-lives of IgG antibodies are influenced by their strong affinity for the neonatal Fc receptor at pH 6.0–6.5 and their weak (almost negligible) affinity at pH 7.0–7.5. This characteristic protects IgG antibodies from degradation within endosomes and enables them to be recycled back into the plasma. The introduction of specific mutations into the Fc-binding region of satralizumab increases its plasma half-life by increasing its affinity for the neonatal Fc receptor, thereby enabling the use of lower doses and longer intervals between doses when compared with other anti–IL-6/IL-6R mAbs, potentially reducing treatment burden [54, 123, 130]. Satralizumab has been formulated for subcutaneous administration to offer a more convenient treatment option for individuals with TED requiring long-term therapy.

The safety and efficacy of satralizumab in individuals with TED who are either treatment-naïve or previously treated with glucocorticosteroids are currently being evaluated in two identically designed, phase 3, multicenter trials called SatraGO-1 and SatraGO-2 (NCT05987423 and NCT06106828, respectively). These trials include approximately 240 participants with active, moderate-to-severe TED or chronic, inactive TED who are receiving treatment with subcutaneous satralizumab or placebo every 2 weeks for the first three doses, followed by every 4 weeks. Participants who show responses in evaluations of proptosis at week 24 will be re-randomized to either continue treatment with satralizumab or receive placebo from week 24 to week 48. The primary efficacy endpoint of both studies, which are due to complete in 2026, is the proportion of participants who achieve ≥ 2-mm reduction in proptosis of the study eye from baseline to week 24.

### Pacibekitug

Pacibekitug is an investigational, human anti–IL-6 mAb developed to treat individuals with active, moderate-to-severe TED and atherosclerotic cardiovascular disease. Unlike mAbs against IL-6R such as tocilizumab and satralizumab, which block all three IL-6–mediated signaling pathways (cis-, trans-, and trans-presentation signaling), pacibekitug specifically targets IL-6, antagonizing only the cis- and trans-signaling pathways, and may therefore be less potent than IL-6R inhibitors. Pharmacokinetic and pharmacodynamic modeling based on studies of pacibekitug in healthy volunteers and individuals with autoimmune conditions other than TED suggests that doses of 20–50 mg every 8 weeks should provide robust anti-inflammatory effects in individuals with TED [131]. The safety and efficacy of 20-mg and 50-mg doses of pacibekitug every 8 weeks are being compared with placebo in the ongoing phase 2b spiriTED trial (NCT06088979). The primary efficacy endpoint of this study involving 81 participants is the reduction in proptosis or abnormal eye protrusion at week 20, with initial data expected in early 2025.

### Sarilumab

Sarilumab is a human anti–IL-6R mAb approved for the treatment of rheumatoid arthritis [44]. Results from a descriptive, retrospective case series of individuals with active, moderate-to-severe TED previously treated with glucocorticosteroids and subsequently treated with either tocilizumab (*n* = 15) or sarilumab (*n* = 5) suggest that sarilumab may be well tolerated and effective [132]. This small case series reported a mean reduction in CAS of 3 points after 3–6 months of treatment, with all participants achieving inactive disease and reductions in disease severity. Improvements in parameters describing thyroid function and reductions in the titers of anti–TSH-R and anti–thyroid peroxidase autoantibodies were also reported. At the time of follow-up, all but one participant (who had discontinued due to clinical remission) remained on treatment. None required subsequent immunosuppressive therapy or surgery. The only AE reported was a single case of mild neutropenia. To our knowledge, the safety and efficacy of sarilumab in individuals with TED have not been evaluated in an RCT.

### Other anti–IL-6/IL6R mAbs

To our knowledge, no clinical studies of vobarilizumab, levilimab, olokizumab, sirukumab, clazakizumab or siltuximab have been conducted in individuals with TED.

## Conclusions

Recent evidence supporting the role of IL-6–mediated signaling in the pathogenesis of TED has generated considerable interest in the use of anti–IL-6/IL-6R mAbs for the treatment of individuals with moderate-to-severe disease. Tocilizumab was the first anti–IL-6R mAb to show meaningful reduction of disease activity alongside improvements in proptosis and ocular motility in patients with TED. As a result, even with limited clinical trial data, tocilizumab was included as second-line treatment in the EUGOGO 2021 guidelines and the joint ATA and ETA consensus, despite not having approval for the treatment of TED. Emerging investigational anti–IL-6/IL-6R mAbs, such as satralizumab and pacibekitug, may offer a more effective and convenient alternative for subcutaneous administration in active, moderate-to-severe TED, pending results of ongoing trials.

## Supplementary Information

Below is the link to the electronic supplementary material.

Supplementary File 1 (DOCX 25.1 KB)

## Data Availability

No datasets were generated or analysed during the current study.

## Abbreviations

ADAM17: A disintegrin and metalloprotease domain 17
AE: Adverse event
ATA: American Thyroid Association
CAS: Clinical Activity Score
EUGOGO: European Group On Graves’ Orbitopathy
ETA: European Thyroid Association
IGF-1: Insulin-like growth factor 1
IGF-1R: Insulin-like growth factor 1 receptor
IgG2: Immunoglobulin G2
IL-6: Interleukin 6
IL-6R: Interleukin 6 receptor
IL-6Rα: Interleukin 6 receptor alpha
JAK: Janus kinase
mIL-6Rα: Membrane-bound interleukin 6 receptor alpha
mRNA: Messenger RNA
RANTES: Regulated upon activation, normal T cell expressed and secreted
RCT: Randomized controlled trial
sIL-6Rα: Soluble interleukin 6 receptor alpha
SOCS3: Suppressor of cytokine signaling 3
STAT: Signal transducer and activator of transcription
TED: Thyroid eye disease
TGF-β: Transforming growth factor beta
Th17: T helper 17
TNF-α: Tumor necrosis factor alpha
Treg: Regulatory T cell
TSH-R: Thyroid-stimulating hormone receptor
VEGF: Vascular endothelial growth factor
