# Supplementary material for: The role of monoclonal antibodies against IL-6 or IL-6R in the treatment of thyroid eye disease
Source: Rev Endocr Metab Disord. 2026 Feb 16;27(2):237–53. doi: 10.1007/s11154-025-10014-5 (PMC13167855; doi:10.1007/s11154-025-10014-5)
Supplement: Supplementary file 1 — (DOCX 25.1 KB) [file 11154_2025_10014_MOESM1_ESM.docx]

**Publications for Daniel Ezra, M.D.**

(past 10 years, those related to thyroid eye disease are in blue)

1. Kok LT, Malik M, Fayers T, Sorkou K, Issa A, **Ezra DG.** Linear Scleroderma En Coup de Sabre: A Case Series With Eyelid Involvement and Management. Ophthalmic Plast Reconstr Surg. 2025;41(3):e80-e5.

2. Wilde CL, Jiang K, Lee S, **Ezra DG.** The Posthyaluronidase Syndrome: Dosing Strategies for Hyaluronidase in the Dissolving of Facial Filler and Independent Predictors of Poor Outcomes. Plast Reconstr Surg Glob Open. 2024;12(4):e5765.

3. Wilde CL, **Ezra DG.** Orbital compartment syndrome following tear trough filler injection. Orbit. 2024;43(3):389-91.

4. Ting MA, Manta AI, Samia-Aly E, Lai M, de Carvalho ER, Buttery P, et al. Blepharospasm Secondary to Deep Brain Stimulation of the Subthalamic Nucleus in Parkinson Disease: Clinical Characteristics and Management Outcomes. J Neuroophthalmol. 2024;44(4):517-22.

5. Parker R, Kaushik M, Bansal O, **Ezra DG.** Safety and Efficacy of Mucograft Porcine Collagen Bioengineered Acellular Dermal Matrix as a Spacer Graft in Lower Eyelid Elevation Surgery. Plast Reconstr Surg Glob Open. 2024;12(1):e5562.

6. Murta F, Guevara GF, Hyer JN, **Ezra DG.** Quantitative Assessment of Periocular Autologous Fat Transfer Survival Using 3D Stereophotogrammetric Imaging. Ophthalmic Plast Reconstr Surg. 2024;40(1):55-60.

7. Memon SF, Wilde CL, **Ezra DG.** Lower Eyelid Surgical Anatomy and the Implications for Blepharoplasty Surgery: A Systematic Review of Anatomic Studies in the Literature. J Craniofac Surg. 2024.

8. Mathewson PA, Athwal S, Hyer JN, **Ezra DG.** The Whitnall Barrier: A Technique for the Management of Lacrimal Gland Prolapse. Plast Reconstr Surg. 2024;153(1):216-20.

9. Wilde CL, Gupta A, Lee S, **Ezra DG.** Tear Trough Filler Using the Three-point Tangent Technique: Lessons from 1452 Tear Trough Applications. Plast Reconstr Surg Glob Open. 2023;11(6):e5060.

10. Wilde C, Kaushik M, Neumann I, Verma P, Azzopardi EA, **Ezra DG.** Safety and efficacy of subcutaneous diode laser as a novel treatment in the management of festoons. Lasers Med Sci. 2023;38(1):170.

11. Ting MAJ, **Ezra DG.** The Tear Trough and Lower Lid Folds: Etiology and Implications for Treatment. Int Ophthalmol Clin. 2023;63(3):13-33.

12. Kaushik M, Juniat V, **Ezra DG**, Verity DH, Uddin J, Timlin H**.** Blood-stained tears-a red flag for malignancy? Eye (Lond). 2023;37(8):1711-6.

13. Biscarini F, Masetti G, Muller I, Verhasselt HL, Covelli D, Colucci G, et al. Gut Microbiome Associated With Graves Disease and Graves Orbitopathy: The INDIGO Multicenter European Study. J Clin Endocrinol Metab. 2023;108(8):2065-77.

14. Ah-Kye L, Butt A, Gupta A, Timlin H, Daniel C, Uddin J, et al. Introducing the 'Benign Eyelid Lesion Pathway': 1 year experience of synchronous tele-oculoplastics in a tertiary hospital. Eye (Lond). 2023;37(7):1458-63.

15. Ting M, **Ezra DG.** Unravelling the Complex Anatomy of the Tear Trough and Lower Eyelid Folds: A Review of Cadaveric Studies in the Literature. J Craniofac Surg. 2022;33(8):2670-6.

16. Lawes-Wickwar S, McBain H, Brini S, Hirani SP, Hurt CS, Flood C, et al. A patient-initiated treatment model for blepharospasm and hemifacial spasm: a randomized controlled trial. BMC Neurol. 2022;22(1):99.

17. Kang S, Raja L, Sim DA, Thomas PBM, **Ezra DG.** Telemedicine in oculoplastic and adnexal surgery: clinicians' perspectives in the UK. Br J Ophthalmol. 2022;106(10):1344-9.

18. Kang S, Hamed Azzam S, Minakaran N, **Ezra DG.** Rituximab for thyroid-associated ophthalmopathy. Cochrane Database Syst Rev. 2022;6(6):Cd009226.

19. Timlin HM, Kang S, Jiang K, **Ezra DG.** Recurrent epiphora after dacryocystorhinostomy surgery: Structural abnormalities identified with dacryocystography and long term outcomes of revision surgery : Success rates of further surgery following failed dacryocystorhinostomy surgery. BMC Ophthalmol. 2021;21(1):117.

20. Timlin HM, Jiang K, **Ezra DG.** Impact of Upper Eyelid Surgery on Symptom Severity and Frequency in Benign Essential Blepharospasm. J Mov Disord. 2021;14(1):53-9.

21. Murta F, Hyer JN, Haridas A, Rose GE, **Ezra DG.** Quantitative Assessment of Orbital Decompression Surgery Using Photogrammetric Stereoimaging. Ophthalmic Plast Reconstr Surg. 2021;37(5):420-3.

22. Lawes-Wickwar S, McBain H, Hirani SP, Hurt CS, Dunlop N, Solly D, et al. Which factors impact on quality of life for adults with blepharospasm and hemifacial spasm? Orbit. 2021;40(2):110-9.

23. Hyer JN, Murta F, Juniat VAR, **Ezra DG.** Validating three-dimensional imaging for volumetric assessment of periorbital soft tissue. Orbit. 2021;40(1):9-17.

24. Ting M, **Ezra DG.** Teprotumumab: a disease modifying treatment for graves' orbitopathy. Thyroid Res. 2020;13:12.

25. Timlin HM, Jiang K, Mathewson P, Manta A, Rubinstein T, **Ezra DG.** Long-Term Outcomes of StopLoss Jones Tubes for Epiphora in Patients With Early or Multiple Loss of Lester Jones Tubes. Ophthalmic Plast Reconstr Surg. 2020;36(2):127-31.

26. Taylor PN, Zhang L, Lee RWJ, Muller I, **Ezra DG**, Dayan CM, et al. New insights into the pathogenesis and nonsurgical management of Graves orbitopathy. Nat Rev Endocrinol. 2020;16(2):104-16.

27. Murta FR, Waxman J, Skilton A, Wickwar S, Bonstein K, Cable R, et al. The first UK national blepharospasm patient and public involvement day; identifying priorities. Orbit. 2020;39(4):233-40.

28. Minakaran N, **Ezra DG**, Allan BD**.** Topical anaesthesia plus intracameral lidocaine versus topical anaesthesia alone for phacoemulsification cataract surgery in adults. Cochrane Database Syst Rev. 2020;7(7):Cd005276.

29. Mak FHW, Ting M, Edmunds MR, Harker A, Edirisinghe M, Duggineni S, et al. Videographic Analysis of Blink Dynamics following Upper Eyelid Blepharoplasty and Its Association with Dry Eye. Plast Reconstr Surg Glob Open. 2020;8(7):e2991.

30. Kozdon K, Caridi B, Duru I, **Ezra DG**, Phillips JB, Bailly M**.** A Tenon's capsule/bulbar conjunctiva interface biomimetic to model fibrosis and local drug delivery. PLoS One. 2020;15(11):e0241569.

31. Yang IH, Rose GE, **Ezra DG**, Bailly M**.** Macrophages promote a profibrotic phenotype in orbital fibroblasts through increased hyaluronic acid production and cell contractility. Sci Rep. 2019;9(1):9622.

32. Timlin HM, Keane PA, **Ezra DG.** Characterizing Congenital Double Punctum Anomalies: Clinical, Endoscopic, and Imaging Findings. Ophthalmic Plast Reconstr Surg. 2019;35(6):549-52.

33. Lorenzano D, Tansley S, **Ezra DG.** Sensory Trick Frames: A New Device for Blepharospasm Patients. J Mov Disord. 2019;12(1):22-6.

34. Din N, Vasquez-Perez A, **Ezra DG**, Tuft SJ**.** Serious corneal complications and undiagnosed floppy eyelid syndrome; A case series and a 10-year retrospective review. J Curr Ophthalmol. 2019;31(2):225-8.

35. Wickwar S, McBain H, Edmunds MR, **Ezra DG**, Rose GE, Newman SP**.** Patients' expectations for the functional and psychosocial outcomes of orbital decompression surgery for thyroid eye disease: a qualitative study. Psychol Health Med. 2018;23(4):475-84.

36. Timlin HM, Keane PA, Rose GE, **Ezra DG.** Characterizing the Occluded Lacrimal Punctum Using Anterior Segment Optical Coherence Tomography. Ophthalmic Plast Reconstr Surg. 2018;34(1):26-30.

37. Rajendram R, Taylor PN, Wilson VJ, Harris N, Morris OC, Tomlinson M, et al. Combined immunosuppression and radiotherapy in thyroid eye disease (CIRTED): a multicentre, 2 × 2 factorial, double-blind, randomised controlled trial. Lancet Diabetes Endocrinol. 2018;6(4):299-309.

38. Hamed Azzam S, Kang S, Salvi M, **Ezra DG.** Tocilizumab for thyroid eye disease. Cochrane Database Syst Rev. 2018;11(11):Cd012984.

39. Bladen JC, Wang J, Sangaralingam A, Moosajee M, Fitchett C, Chelala C, et al. MicroRNA and transcriptome analysis in periocular Sebaceous Gland Carcinoma. Sci Rep. 2018;8(1):7531.

40. Bladen JC, Gill JS, Miszkiel K, **Ezra DG.** Stroke-induced resolution of primary blepharospasm: evidence for the lenticular nucleus as a control candidate. BMJ Case Rep. 2018;2018.

41. Timlin HM, Keane PA, Rose GE, **Ezra DG.** The Application of Infrared Imaging and Optical Coherence Tomography of the Lacrimal Punctum in Patients Undergoing Punctoplasty for Epiphora. Ophthalmology. 2017;124(6):910-7.

42. Smith TJ, Kahaly GJ, **Ezra DG**, Fleming JC, Dailey RA, Tang RA, et al. Teprotumumab for Thyroid-Associated Ophthalmopathy. N Engl J Med. 2017;376(18):1748-61.

43. Roos JC, **Ezra DG**, Rose GE**.** 'Preoperative imaging should be performed for all cases of acquired nasolacrimal duct obstruction'-No. Eye (Lond). 2017;31(3):349-50.

44. Mellington FE, Dayan CM, Dickinson AJ, Hickey JL, MacEwen CJ, McLaren J, et al. Management of thyroid eye disease in the United Kingdom: A multi-centre thyroid eye disease audit. Orbit. 2017;36(3):159-69.

45. Kwon KA, Shipley RJ, Edirisinghe M, Best SM, Cameron RE, Poitelea C, et al. The Mechanics of Brow-Suspension Ptosis Repair: A Comparative Study of Fox Pentagon and Crawford Triangle Techniques. Ophthalmic Plast Reconstr Surg. 2017;33(1):22-6.

46. Wickwar S, McBain H, Newman SP, Hirani SP, Hurt C, Dunlop N, et al. Effectiveness and cost-effectiveness of a patient-initiated botulinum toxin treatment model for blepharospasm and hemifacial spasm compared to standard care: study protocol for a randomised controlled trial. Trials. 2016;17(1):129.

47. Waxman J, Hersh D, Murta F, **Ezra DG.** Language modulation of benign essential blepharospasm. Mov Disord. 2016;31(5):764-5.

48. Timlin HM, Keane PA, Day AC, Salam T, Abdullah M, Rose GE, et al. Characterizing the lacrimal punctal region using anterior segment optical coherence tomography. Acta Ophthalmol. 2016;94(2):154-9.

49. McBain HB, MacKenzie KA, Hancox J, **Ezra DG**, Adams GG, Newman SP**.** Does strabismus surgery improve quality and mood, and what factors influence this? Eye (Lond). 2016;30(5):656-67.

50. McBain H, MacKenzie K, Hancox J, **Ezra DG**, Adams GG, Newman SP**.** What do patients with strabismus expect post surgery? The development and validation of a questionnaire. Br J Ophthalmol. 2016;100(3):415-9.

51. Mak FH, Harker A, Kwon KA, Edirisinghe M, Rose GE, Murta F, et al. Analysis of blink dynamics in patients with blepharoptosis. J R Soc Interface. 2016;13(116).

52. MacKenzie K, Hancox J, McBain H, **Ezra DG**, Adams G, Newman S**.** Psychosocial interventions for improving quality of life outcomes in adults undergoing strabismus surgery. Cochrane Database Syst Rev. 2016;2016(5):Cd010092.

53. Kechagia JZ, **Ezra DG**, Burton MJ, Bailly M**.** Fibroblasts profiling in scarring trachoma identifies IL-6 as a functional component of a fibroblast-macrophage pro-fibrotic and pro-inflammatory feedback loop. Sci Rep. 2016;6:28261.

54. Casswell EJ, Salam T, Sullivan PM, **Ezra DG.** Ophthalmology trainees' self-assessment of cataract surgery. Br J Ophthalmol. 2016;100(6):766-71.

55. Adams GG, McBain H, MacKenzie K, Hancox J, **Ezra DG**, Newman SP**.** Is strabismus the only problem? Psychological issues surrounding strabismus surgery. J aapos. 2016;20(5):383-6.

56. Wickwar S, McBain H, **Ezra DG**, Hirani SP, Rose GE, Newman SP**.** The Psychosocial and Clinical Outcomes of Orbital Decompression Surgery for Thyroid Eye Disease and Predictors of Change in Quality of Life. Ophthalmology. 2015;122(12):2568-76.e1.

57. Perros P, Dayan CM, Dickinson AJ, **Ezra DG**, Hickey JL, Hintschisch C, et al. Future Research in Graves' Orbitopathy: From Priority Setting to Trial Design Through Patient and Public Involvement. Thyroid. 2015;25(11):1181-4.

58. Fayers T, Shaw SR, Hau SC, **Ezra DG.** Changes in corneal aesthesiometry and the sub-basal nerve plexus in benign essential blepharospasm. Br J Ophthalmol. 2015;99(11):1509-13.
